# Complement abnormality predisposes to the development of malignant hypertension-associated thrombotic microangiopathy disease

**DOI:** 10.1093/ckj/sfaf235

**Published:** 2025-07-24

**Authors:** Rong Lian, Wenchuan Li, Yuejiao Li, Xinji Lian, Shengyou Yu, Wanxin Shi, Jianwen Yu, Wei Chen, Jianbo Li, Feng He

**Affiliations:** Department of Nephrology, Guangzhou First People's Hospital, The Second Affiliated Hospital, School of Medicine, South China University of Technology, Guangzhou, China; Department of Clinical Medical Research, Guangzhou First People's Hospital, The Second Affiliated Hospital, School of Medicine, South China University of Technology, Guangzhou, China; Department of Nephrology, Guangzhou First People's Hospital, The Second Affiliated Hospital, School of Medicine, South China University of Technology, Guangzhou, China; Department of Nephrology, Guangzhou First People's Hospital, The Second Affiliated Hospital, School of Medicine, South China University of Technology, Guangzhou, China; Department of Clinical Medical Research, Guangzhou First People's Hospital, The Second Affiliated Hospital, School of Medicine, South China University of Technology, Guangzhou, China; Department of Geriatrics, Guangzhou First People's Hospital, The Second Affiliated Hospital, School of Medicine, South China University of Technology, Guangzhou, China; Department of Clinical Medical Research, Guangzhou First People's Hospital, The Second Affiliated Hospital, School of Medicine, South China University of Technology, Guangzhou, China; Department of Nephrology, Guangzhou First People's Hospital, The Second Affiliated Hospital, School of Medicine, South China University of Technology, Guangzhou, China; Department of Nephrology, The First Affiliated Hospital, Sun Yat-sen University, NHC Key Laboratory of Clinical Nephrology (Sun Yat-sen University) and Guangdong Provincial Key Laboratory of Nephrology, Guangzhou, China; Department of Nephrology, The First Affiliated Hospital, Sun Yat-sen University, NHC Key Laboratory of Clinical Nephrology (Sun Yat-sen University) and Guangdong Provincial Key Laboratory of Nephrology, Guangzhou, China; Department of Nephrology, The First Affiliated Hospital, Sun Yat-sen University, NHC Key Laboratory of Clinical Nephrology (Sun Yat-sen University) and Guangdong Provincial Key Laboratory of Nephrology, Guangzhou, China; Department of Nephrology, Guangzhou First People's Hospital, The Second Affiliated Hospital, School of Medicine, South China University of Technology, Guangzhou, China

**Keywords:** complement abnormality, kidney biopsy, malignant hypertension, renal prognosis, thrombotic microangiopathy

## Abstract

**Background:**

Thrombotic microangiopathy (TMA) is a major complication of malignant hypertension (mHTN). Abnormal complement activation has been recognized as a key determinant of TMA, but less is known about the prognostic significance of complement abnormality in patients with mHTN-associated TMA.

**Methods:**

A prospective cohort study was performed in patients with mHTN. All participants had concomitant TMA proven by kidney biopsy after admission between 2008 and 2023, and were divided into normal and abnormal complement groups based on serum C3 and C4 levels. Cox regression models were used to identify risk factors for renal prognosis.

**Results:**

A total of 189 mHTN patients with TMA were enrolled in the current study, including 161 (85.2%) patients with normal complement levels and 28 (14.8%) patients with abnormal complement levels. Compared to the normal complement group, patients in the abnormal complement group had lower levels of BMI, hemoglobin, and platelet counts, and more intravascular erythrocyte fragments (21.4% vs 7.5%, *P* = .02). Notably, a substantial glomerular deposition of C3c and C5b-9 was observed in the abnormal complement group, indicating complement activation *in vivo*. Importantly, abnormal complement levels were independently associated with worse renal function recovery [hazard ratio (HR), 0.368; 95% CI, 0.140–0.970; *P* = .043]. In addition, the glomerular sclerosis ratio (HR, 0.971; 95% CI, 0.953–0.989; *P* = .002) remained an independent predictor of poor renal outcomes.

**Conclusions:**

Patients with abnormal complement levels have worse renal prognosis, suggesting that complement abnormality predisposes to the progression of mHTN-associated TMA disease.

## Graphical Abstract

**Graphical Abstract:**
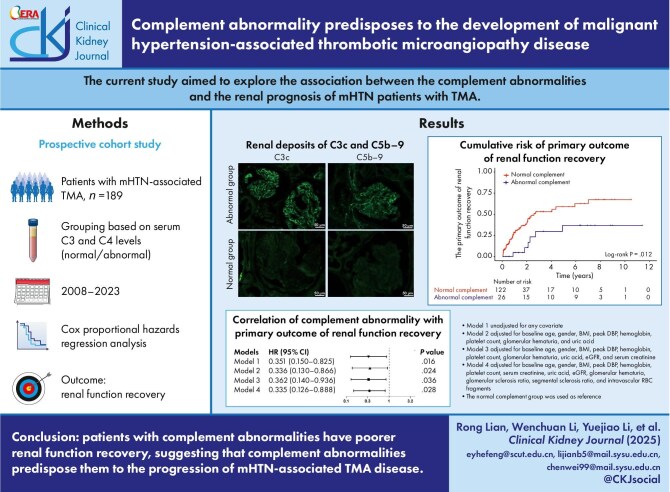

KEY LEARNING POINTS **What was known:**

- Thrombotic microangiopathy (TMA) is a major complication of malignant hypertension (mHTN).
- Although accumulating evidence links abnormal complement activation to various TMA syndromes, its impact on the prognosis of patients with mHTN-associated TMA remains unclear.

**This study adds:**

- Patients in the abnormal complement group had lower levels of BMI, hemoglobin, and platelet counts, and more intravascular erythrocyte fragments.
- Renal deposits of C5b-9 and C3c were observed in the abnormal complement group.
- Abnormal complement levels were independently associated with worsening of the renal function recovery.

**Potential impact:**

- These findings highlighted the prognostic value of complement abnormality on renal outcomes and provided guidance for clinical practice in mHTN patients.
- It is imperative to screen for complement abnormality in patients with mHTN-associated TMA, as this will facilitate the potential use of complement inhibitors in conjunction with conventional antihypertensive therapy.

## INTRODUCTION

Malignant hypertension (mHTN) is characterized by a sudden and large increase in blood pressure (BP), especially diastolic BP [1]. It is associated with widespread endothelial damage and acute ischemic organ damage, including grade Ⅲ/Ⅳ retinopathy, renal failure, left ventricular dysfunction, central neurological damage, and so on [1–5]. Although its prognosis has improved remarkably in recent decades after the administration of modern antihypertensive drugs, acute complications and chronic sequelae are common, with the kidney being one of the major organs affected [6–8].

Traditionally, it was commonly accepted that mHTN may induce direct damage to the vascular endothelium, thus ultimately leading to thrombotic microangiopathy (TMA) [5, 9]. TMA presents as a complication of mHTN [10] and its prevalence in mHTN ranges from 14% to 46% [6]. In fact, 16%–28% of patients with mHTN-associated TMA develop end-stage renal disease (ESRD) requiring dialysis or kidney transplantation within 5–10 years [8]. In addition, mHTN-associated TMA is likely to lead to renal graft failure or destruction [11]. A better understanding of the pathogenesis of mHTN-associated TMA may improve these adverse outcomes. However, the underlying mechanisms of mHTN-associated TMA are still incompletely understood.

Malignant hypertension-associated TMA is characterized by diffuse capillary loop wrinkling and capsule thickening, marked renal artery intimal thickening, vessel wall thickening with “onion skin lesion” appearance, fibrinoid necrosis, and intravascular thrombosis, among others [11], which probably lead to end-organ ischemia and infarction, especially affecting the kidney and brain [5]. To explain mHTN-induced TMA, accumulating evidence suggests that the extremely high BP induces direct damage to the vascular endothelium and leads to the profound pathological events of TMA. Based on this pathogenic hypothesis, BP control alone would reverse TMA and other complications. Unfortunately, deterioration of renal function is still observed in a significant proportion (14%) of TMA patients despite intensive BP management. It is suggested that other pathogenic factors involved in the pathogenesis of mHTN-associated TMA urgently need to be elucidated.

Until recently, aberrant activation of the complement alternative pathway is a cardinal feature of TMA [12, 13]. Mutations in complement-related genes that either regulate or activate the complement alternative pathway have been identified in six of nine mHTN patients with TMA [11]. Elevated plasma C5b-9 levels and renal deposition of C3c and C5b-9 along glomerular capillary walls have also been detected in these patients [11]. However, whether complement abnormality had an important effect on renal outcomes in patients with mHTN-associated TMA is still unclear.

Therefore, the current study aimed to explore the association between the clinicopathological characteristics and renal prognosis of mHTN patients with TMA using a prospective cohort study. The study highlighted the prognostic value of complement abnormality and provided guidance for clinical practice in mHTN patients.

## MATERIALS AND METHODS

### Study design and patients

This observational cohort study consecutively enrolled mHTN patients with renal biopsy-proven TMA diagnosed at The First Affiliated Hospital of Sun Yat-sen University from January 2008 to June 2023. The eligibility criteria for the cohort study were: (i) patients aged ≥18 years; (ii) patients met the diagnostic criteria for mHTN; and (iii) a renal biopsy confirmed the presence of renal TMA. It represents the renal manifestation of malignant hypertension and is pathologically defined by two hallmark features: (i) fibrinoid necrosis of arterioles, necrotizing damage to arterioles, mediated by endothelial injury and fibrin deposition, and leads to luminal obliteration, ischemia, and glomerular collapse; and (ii) hyperplastic arteriolosclerosis of onion-peel appearance, concentric intimal thickening of small arteries (e.g. interlobular arteries) due to smooth muscle cell proliferation, and extracellular matrix deposition. In addition, the exclusion criteria for atypical hemolytic uremic syndrome (aHUS) patients with malignant hypertension in our cohort were: (i) patients with concomitant glomerulonephritis, immunoglobulin A (IgA) nephropathy, and benign hypertension accompanied by aHUS; and (ii) patients with malignant hypertension and renal TMA caused by other aHUS related diseases, such as malignant tumors, pregnancy, drug toxicity, renal transplantation, rheumatic immune diseases (systemic lupus erythematosus, antiphospholipid syndrome, etc.), and viral infections. This study adhered to the ethical standards of the Declaration of Helsinki and was approved by the Ethics Committee of the First Affiliated Hospital of Sun Yat-sen University. Written informed consent was obtained from all patients before enrollment.

### Definitions

The clinical diagnosis of mHTN is characterized by a rapid increase in arterial BP, with diastolic BP (DBP) exceeding 130 mmHg, accompanied by grade III or IV hypertensive retinopathy according to the Keith–Wagener classification and other evidence of impending target organ dysfunction secondary to hypertension [14, 15]. TMA was diagnosed by a renal pathologist and a nephrologist based on both clinical presentation and renal pathological features. Renal histopathological lesions in TMA tend to be characterized by various pathological changes such as capillary loop wrinkling, capsule thickening, significant renal artery intimal thickening, “onion skin” vessel wall thickening, fibrinoid necrosis, intravascular thrombosis, ischemic glomerular changes, and tubular necrosis [5, 11, 16].

### Clinical characteristics, laboratory data, and renal histopathology

Medical records were used to obtain clinical characteristics and laboratory data, including age, sex, body mass index (BMI), smoking and drinking status, and BP on admission and discharge. Medication use was also recorded, including angiotensin-converting enzyme inhibitors, angiotensin receptor blockers or sacubitril/valsartan (ARNIs), α-blockers, β-blockers, calcium channel blockers, statins, sulodexide, etc.

Laboratory data at the time of biopsy were also obtained from medical records, including platelet count, hemoglobin, serum albumin, lipid index, serum creatinine, uric acid, serum C3 and C4, and 24-hour proteinuria levels. Serum C3 and C4 levels were measured at the time of diagnosis using a turbidimetric assay. Our laboratory's reference range for serum C3 is 0.79–1.17 g/l, and the reference range for serum C4 is 0.17–0.31 g/l. The normal complement group was recognized as a serum C3 level between 0.79 and 1.17 g/l, and the abnormal group was defined as a serum C3 level below 0.79 g/l with a normal serum C4 level.

Kidney biopsies were processed for immunofluorescence, light, and electron microscopy as described [17]. The study collected data on several light microscopic parameters, including glomerular number, global sclerosis and segmental sclerosis. Tubulointerstitial parameters, such as tubular atrophy/interstitial fibrosis and tubular epithelial cell sloughing, were also recorded. In addition, vascular parameters including fibrous necrosis, onion skin lesions, intravascular thrombosis, and intravascular erythrocyte fragments were documented. Electron microscopic evaluation included identification of subepithelial, subendothelial, or mesangial deposits and assessment of the degree of endothelial cell swelling. Patients with confirmed concomitant renal disease or inadequate biopsy for diagnosis were excluded.

### Detection of renal C3c and C5b-9 by immunofluorescence

For direct immunofluorescence, 3-μm frozen sections were analyzed for deposition of C3c and C5b-9 using rabbit anti-C3c mAb (1:300 dilution; 66157–1-Ig, Proteintech) and rabbit anti-C5b-9 mAb (1:250 dilution; bs-2673R, Bioss) as primary antibodies. Fluorescein isothiocyanate-conjugated anti-rabbit Ab (1:100 dilution; Zhongshan Golden Bridge) was used as a secondary antibody. After incubation with these antibodies, the deposition of C3c and C5b-9 was observed by laser scanning confocal microscopy.

### Study outcomes

The aim of this study was to evaluate the association between complement activation and renal outcome in the cohort of patients with mHTN-associated TMA. The primary outcome of this study was renal function recovery, defined as a >50% decrease in serum creatinine compared to baseline, a decrease in serum creatinine to normal, or renal survival free of hemodialysis or peritoneal dialysis for at least 1 month. All patients were followed up by nephrologists and trained nurses through office visits or telephone interviews, with the last follow-up date being 30 June 2023.

### Statistical analysis

Continuous variables were presented as mean [standard deviation (SD)] or median [interquartile range (IQR)]. Continuous variables were compared using the Mann–Whitney *U*-test for non-normally distributed variables and the Student’s *t*-test for normally distributed variables. Categorical variables were presented as absolute and relative frequencies and percentages, and analyzed with the chi-squared test or Fisher’s exact test. The time to reach study outcomes was assessed using the Kaplan–Meier model. Survival comparisons between the abnormal group and the normal group were conducted using a log-rank test. Crude and adjusted hazard ratios (HRs) with corresponding 95% confidence intervals (CIs) were calculated employing univariate and multivariate Cox proportional hazards regression models to analyze the impact of complement abnormality on outcomes. Moreover, an interaction test was performed to assess whether patient characteristics might influence the relationship between complement status and renal recovery. All analyses were considered statistically significant if the two-tailed *P* value was <.05. All analyses were performed using SPSS version 25.0 (IBM, Armonk, NY, USA).

## RESULTS

### Baseline characteristics of participants

**Figure 1:**
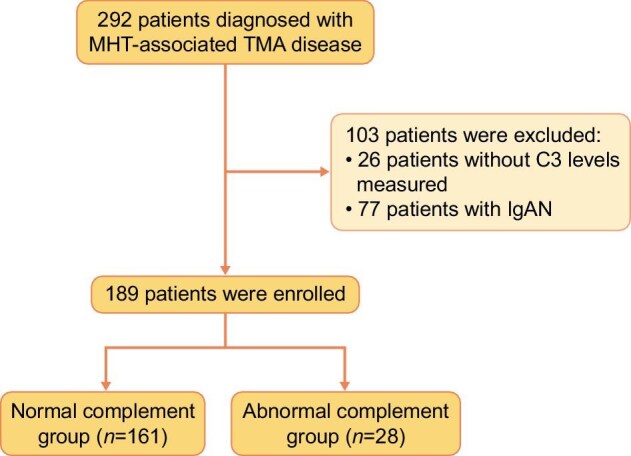
Flow chart of the study. IgAN, IgA nephropathy.

In the study, a total of 189 patients were included and underwent pathological diagnosis for mHTN-associated TMA. The baseline demographic and clinical features of these patients are shown in Table 1. A flowchart illustrating this process is provided in Fig. 1. A total of 161 (85.2%) patients were initially classified into the normal complement group, while 28 (14.8%) patients were categorized into the abnormal complement group based on baseline serum levels. The corresponding scatter plots of serum C3 and C4 levels are presented in Supplementary Fig. S1. The baseline mean age was 37.29 years (SD 8.42). Compared to patients with normal complement, patients with abnormal complement had lower levels of BMI (23.43 ± 2.75 vs 26.06 ± 4.28 kg/m^2^, *P* = .002), hemoglobin (98.46 ± 20.56 vs 109.55 ± 20.84 g/l, *P* = .010), platelet counts (220.18 ± 58.24 vs 280.74 ± 92.27 × 10^9^/l, *P* < .001), eGFR (11.73 ± 7.12 v. 15.94 ± 14.28 ml/min/1.73 m^2^, *P* = .019), and a lower percentage of sulodexide treatment [11 (39.3%) vs 96 (59.6%), *P* = .045]. There were no significant differences in the utilization of other medication between the two groups.

**Table 1:** Baseline characteristics of patients.

		Complement status		
Characteristic	Total (*n* = 189)	Normal (*n* = 161)	Abnormal (*n* = 28)	*P* value
Demographics				
Age, mean (SD), y	37.29 (8.42)	37.51 (8.49)	36.04 (8.04)	.394
Male, *n* (%)	172 (91.0)	145 (90.1)	27 (96.4)	.277
BMI, mean (SD), kg/m²	25.67 (4.19)	26.06 (4.28)	23.43 (2.75)	.002
Smoking, *n* (%)	96 (50.8)	79 (49.1)	17 (60.7)	.255
Drinking, *n* (%)	61 (32.3)	48 (29.8)	13 (46.4)	.083
Baseline BP, mean (SD), mmHg				
Admission SBP, mmHg	162.11 (31.42)	162.25 (32.11)	161.29 (27.63)	.881
Admission DBP, mmHg	101.44 (22.63)	101.93 (22.04)	98.68 (26.06)	.485
Admission MAP, mmHg	121.67 (24.38)	122.04 (24.28)	119.55 (25.26)	.619
Hypertensive retinopathy grading, *n* (%)				
Mild (Grade 0–2)	24 (12.7)	21 (13.0)	3 (10.7)	.733
Severe (Grade 3–4)	165 (87.3)	140 (87.0)	25 (89.3)	
Laboratory indicators				
Hemoglobin, mean (SD), g/l	107.90 (21.11)	109.55 (20.84)	98.46 (20.56)	.010
Serum albumin, mean (SD), g/l	37.40 (4.44)	37.41 (4.47)	37.33 (4.33)	.930
Platelet count, mean (SD), ×10^9^/l	271.77 (90.54)	280.74 (92.27)	220.18 (58.24)	<.001
Serum calcium, mean (SD), mmol/l	2.21 (0.15)	2.22 (0.15)	2.16 (0.15)	.052
Total cholesterol, mean (SD), mmol/l	4.80 (1.31)	4.83 (1.31)	4.63 (1.31)	.459
Triglyceride, mean (SD), mmol/l	1.90 (0.83)	1.92 (0.82)	1.78 (0.88)	.417
LDL-C, mean (SD), mmol/l	3.02 (0.98)	3.05 (0.99)	2.83 (0.86)	.280
HDL-C, mean (SD), mmol/l	1.07 (0.50)	1.07 (0.54)	1.05 (0.23)	.722
Serum creatinine, mean (SD), μmol/l	489.0 (287.36)	477.37 (288.09)	555.86 (278.78)	.183
eGFR, mean (SD), ml/min/1.73 m^2^	15.31 (13.53)	15.94 (14.28)	11.73 (7.12)	.019
Uric acid, median (IQR), μmol/l	464.0 (387.0, 553.0)	464.0 (387.0, 559.0)	450.4 (378.5, 544.5)	.862
24-h proteinuria, mean (SD), g/day	1.66 (1.37)	1.63 (1.31)	1.88 (1.72)	.438
Serum C3, mean (SD), g/l	1.04 (0.24)	1.09 (0.22)	0.73 (0.06)	<.001
Serum C4, mean (SD), g/l	0.31 (0.12)	0.32 (0.12)	0.24 (0.09)	<.001
Glomerular hematuria, *n* (%)	18 (9.5)	16 (9.9)	2 (7.1)	.642
Base medications, *n* (%)				
Sacubitril-valsartan	43 (22.8)	39 (24.2)	4 (14.3)	.247
β-blocker	161 (85.2)	137 (85.1)	24 (85.7)	.932
α-blocker	128 (67.7)	107 (66.5)	21(75.0)	.372
Statin	91 (48.1)	80 (49.7)	11 (39.3)	.309
Sulodexide	107 (56.6)	96 (59.6)	11 (39.3)	.045

Abbreviations: SBP, systolic BP; MAP, mean arterial pressure; LDL-C, low-density lipoprotein.

Continuous variables are expressed as mean (SD), or as median (IQR). Categorical variables are expressed as frequency (%).

### Renal histopathologic characteristics

**Figure 2:**
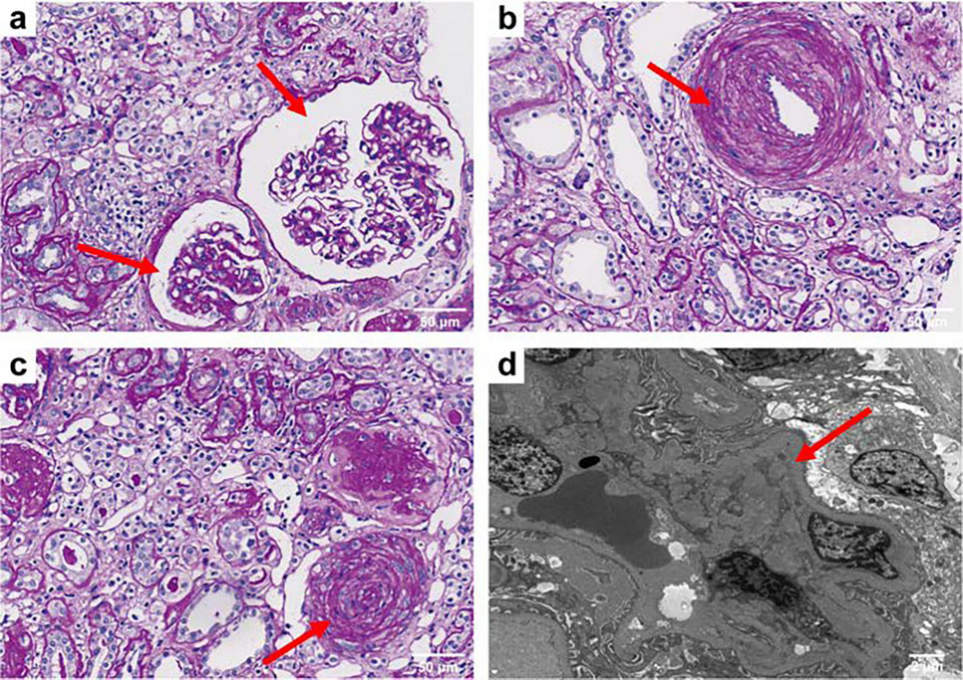
Representative light and electron microscopic findings of malignant hypertension-associated TMA. (**a**) Periodic acid-Schiff staining revealed a widespread wrinkling of the capillary loop. (**b**) Vessel wall thickening with onion skin lesion appearance (red arrow). (**c**) Periodic acid-Schiff staining showing typical intimal thickening and lumen obstruction. (a)–(c), Scale bar: 50 μm. (**d**) Electron microscopy showing endothelial cell swelling alongside pronounced subendothelial expansion characterized by the presence of flocculent material beneath. Scale bar: 2 μm.

All patients diagnosed with mHTN-associated TMA underwent percutaneous renal biopsy. Representative light, electron microscopic, and immunofluorescence findings of mHTN-associated TMA are shown in Fig. 2. Light microscopy examination revealed typical pathological alterations in TMA. These changes encompassed widespread wrinkling of the capillary loops (Fig. 2a), thickening of vessel walls displaying onion skin morphology and luminal occlusion (Fig. 2b), and notable intimal thickening of the renal artery (Fig. 2c). Electron micrographs of a glomerular capillary loop depicted endothelial cell swelling, prominent subendothelial widening with flocculent material deposition, resulting in narrowing of the capillary lumen (Fig. 2d).

**Figure 3:**
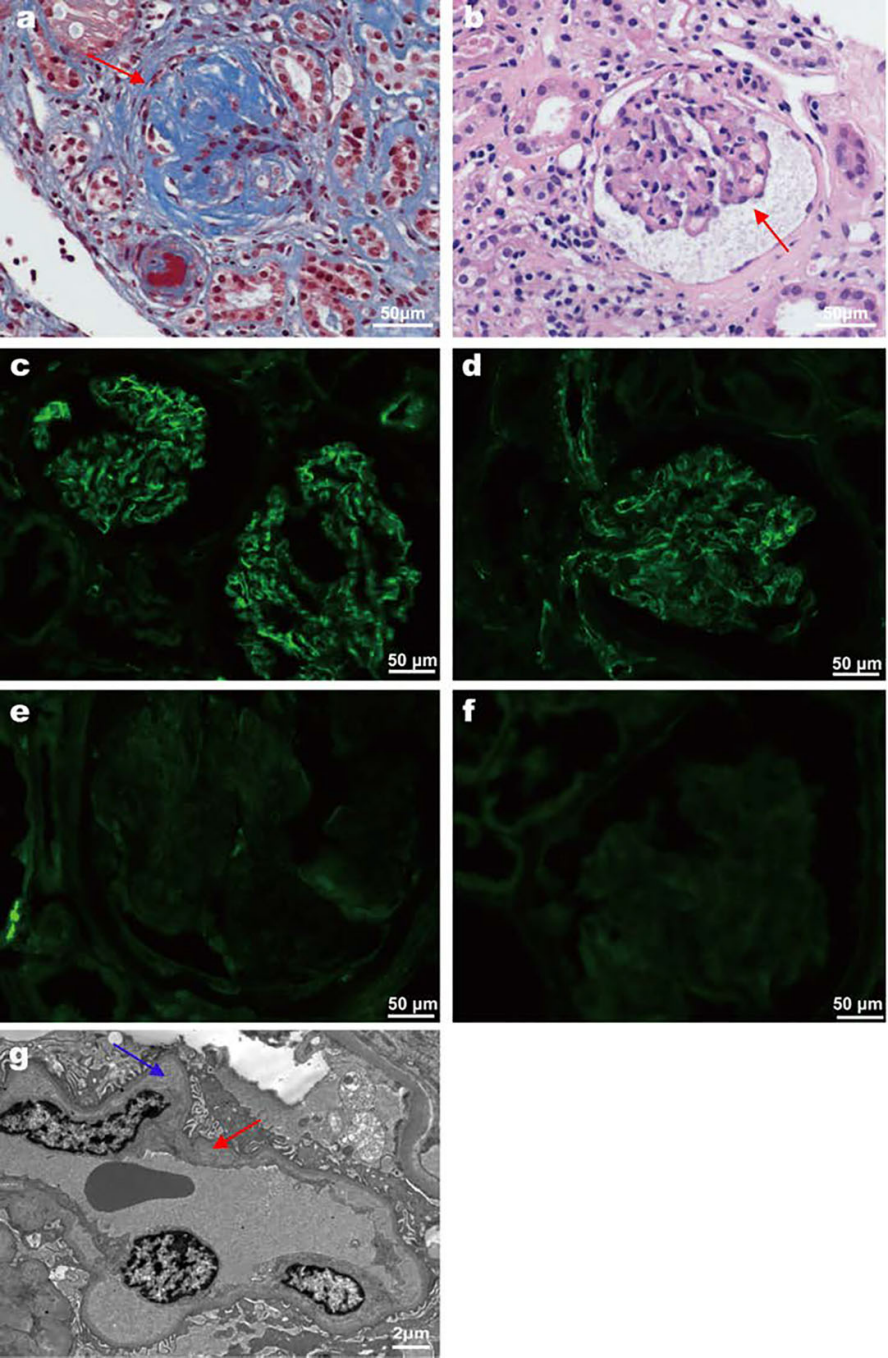
Representative light, immunofluorescence, and electron microscopic findings of malignant hypertension-associated thrombotic microangiopathy (TMA). (**a**) Glomerulosclerosis, microthrombus formation in arterioles and ‘onion skin lesions’ appearance (red arrow) in patients with complement abnormalities (original magnification ×400). (**b**) Glomerular ischemic shrinkage (red arrow), interstitial inflammatory cell infiltration, and protein casts in normal complement group (original magnification ×400). (**c**) C3c (original magnification ×400), and (**d**) C5b-9 (original magnification ×400) deposits along the vasculature and/or the glomerular capillary wall in patients with complement abnormalities. (**e**) C3c (original magnification ×400), and (**f**) C5b-9 (original magnification ×400) deposits along the vasculature and/or the glomerular capillary wall in normal complement group. (**g**) Electron microscopy findings of subendothelial thickening with fibrinoid deposition (blue arrow) and ischemic shrinkage of the basement membrane (red arrow). Scale bars (a–f) 50 μm and (g) 2 μm.

Renal biopsies were subjected to immunostaining for complement components C3c and C5b-9, and their localization was assessed by confocal microscopy. The immunofluorescence findings are shown in Fig. 3. In patients with complement abnormalities, C3c and C5b-9 exhibited predominantly diffuse deposition patterns along the glomerular capillary walls and the vasculature (Fig. 3c and d), with partial segmental distribution (Supplementary Fig. S2), confirming activation of the alternative complement pathway, which is consistent with the findings of Jorge and Timmermans, *et al*. [11, 18]. By contrast, in patients with normal complement levels, C3c and C5b-9 staining was either minimal or below the detection threshold (Fig. 3e and f).

Renal histopathologic findings of patients were shown in Table 2. In comparison to patients with normal complement levels, patients with abnormal complement levels had a significantly higher prevalence of intravascular erythrocyte fragments [6 (21.4%) vs 12 (7.5%), *P* = .020]. There were no statistical differences in the number of cases with other vascular lesions, including glomerular sclerosis, segmental sclerosis, hyaline degeneration, fibrous necrosis, intravascular thrombosis, and tubular atrophy/interstitial fibrosis, across the different groups (*P* > .05).

**Table 2:** Histopathological findings of patients.

	Complement group		
Renal pathology characteristic	Normal (*n* = 161)	Abnormal (*n* = 28)	*P* value
Total glomeruli number, mean (SD)	26.86 (11.90)	25.96 (12.04)	.713
Glomerular sclerosis number, mean (SD)	7.89 (6.16)	9.68 (8.68)	.187
Glomerular sclerosis ratio, mean (SD), %	30.80 (20.24)	38.90 (27.30)	.143
Segmental sclerosis number, mean (SD)	0.93 (1.32)	0.68 (1.09)	.351
Segmental sclerosis ratio, mean (SD), %	3.70 (6.01)	2.86 (4.72)	.483
Vascular parameters (arbitrary units), *n* (%)			
Hyaline degeneration	93 (57.8)	16 (57.1)	.951
Fibrous necrosis	49 (30.4)	10 (35.7)	.578
Onion skin lesions	101 (62.7)	16 (57.1)	.574
Intravascular thrombosis	26 (16.1)	5 (17.9)	.822
Intravascular RBC fragments	12 (7.5)	6 (21.4)	.020
Tubular atrophy/interstitial fibrosis, *n* (%)			.659
<25%	9 (5.6)	0 (0)	
25%–50%	39 (24.4)	7 (25.9)	
50%–75%	96 (60.0)	17 (63.0)	
>75%	16 (10.0)	3 (11.1)	
Tubular epithelial cell exfoliation, *n* (%)	73 (45.3)	9 (32.1)	.193

Abbreviation: RBC, red blood cell.

Continuous variables are expressed as mean (SD). Categorical variables are expressed as frequency (%).

### Risk of complement abnormality on renal function recovery

**Figure 4:**
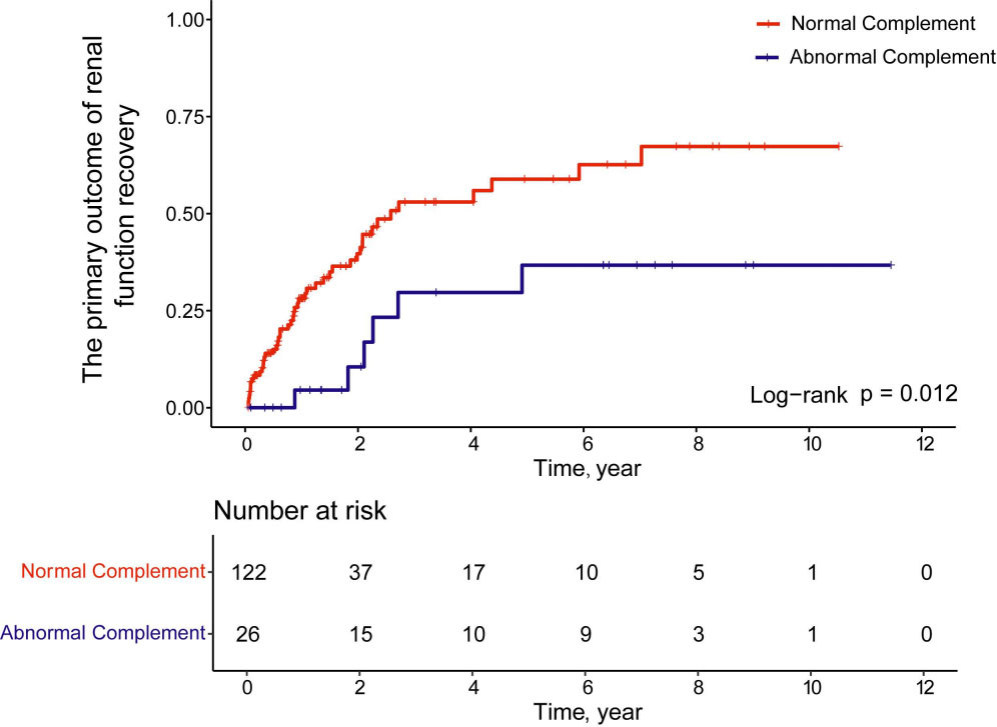
Cumulative risk of primary outcome of renal function recovery in mHTN patients in overall comparison. The primary outcome of this study was defined as a 50% decrease in serum creatinine, or a decrease in serum creatinine to normal, or renal survival free from replacement therapy for at least 1 month.

With a median follow-up period of 12.4 months (IQR, 4.6–29.3), 24 (36.5%) of the primary outcomes occurred. The cumulative effect of complement abnormality on the hazard of the first occurrence of the primary outcome was significantly lower compared to patients with normal complement group (*P* = .012; Fig. 4). In the crude analysis, patients with complement abnormality were significantly associated with worse renal function recovery than those with normal complement levels (HR, 0.351; 95% CI, 0.150–0.825; *P* = .016) (Table 3). In the multivariable Cox regression model adjusting for confounders with a *P* <0.05 in the univariate regression analysis, complement abnormality was significantly associated with poorer renal function recovery compared to patients with normal complement levels (adjusted HR, 0.368; 95% CI, 0.140–0.970; *P* = .043) (Table 3). Additionally, a higher proportion of glomerular sclerosis in a renal biopsy specimen was significantly associated with a lower risk of renal function recovery (adjusted HR, 0.971; 95% CI, 0.953–0.989; *P* = .002) (Table 3).

**Table 3:** Univariable and multivariable Cox regression analysis for the primary outcome of this study.

	Univariate analysis		Multivariate analysis	
Variable	HR (95% CI)	*P* value	HR (95% CI)	*P* value
Complement status (abnormal vs normal)	0.351 (0.150–0.825)	0.016*	0.368 (0.140–0.970)	.043
Age	0.993 (0.962–1.025)	0.685	0.986 (0.951–1.023)	.448
Gender (female vs male)	1.258 (0.453–3.498)	0.659	1.061 (0.347–3.241)	.917
BMI (kg/m^2^)	1.032 (0.964–1.104)	0.366	1.010 (0.924–1.105)	.825
Peak DBP (mmHg)	1.006 (0.992–1.019)	0.412		
Hemoglobin (g/l)	0.996 (0.983–1.009)	0.52	0.996 (0.977–1.015)	.668
Platelet count (10^9^/l)	1.003 (1.000,1.006)	0.055	1.003 (0.999–1.006)	.143
Serum albumin (g/l)	0.962 (0.905–1.023)	0.212		
Glomerular hematuria (yes vs no)	0.577 (0.140–2.371)	0.445	0.605 (0.137–2.663)	.506
Intravascular RBC fragments (%)	1.004 (0.472–2.135)	0.992		
24-h proteinuria (g/day)	0.906 (0.699–1.175)	0.459		
Uric acid (µmol/l)	1.000 (0.997–1.002)	0.862	0.999 (0.996–1.001)	.346
Serum creatinine (µmol/l)	1.000 (1.000–1.001)	0.359	1.000 (0.998–1.001)	.918
eGFR (ml/min/1.73 m^2^)	0.977 (0.946–1.009)	0.161	0.960 (0.910–1.013)	.135
Segmental sclerosis ratio (%)	1.024 (0.977–1.074)	0.325		
Glomerular sclerosis ratio (%)	0.972 (0.955–0.989)	0.001*	0.971 (0.953–0.989)	.002
Sacubitril-valsartan (yes vs no)	2.276 (1.237–4.187)	0.008*	1.604 (0.833–3.086)	.157
Statins (yes vs no)	1.511 (0.880–2.595)	0.135		

Abbreviation: RBC, red blood cell.

The primary outcome of this study was recovery of renal function, defined as a >50% decrease in serum creatinine from baseline, or a decrease in serum creatinine to normal, or renal survival free from hemodialysis or peritoneal dialysis for at least 1 month.

*P value <0.05

### Association between serum complement levels and renal function recovery in patients with mHTN-associated TMA

**Figure 5:**
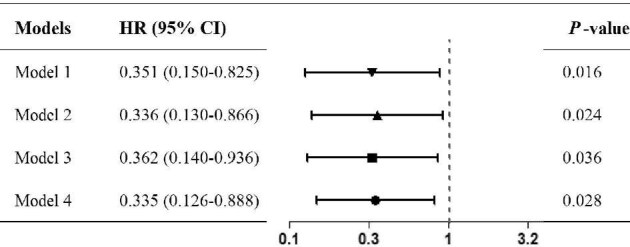
Correlation of the complement abnormality with the primary outcome of renal function recovery. The primary outcome of renal function recovery was defined as a 50% decrease in serum creatinine, or a decrease in serum creatinine to normal, or renal survival free from replacement therapy for at least 1 month. The normal complement group was as reference. Model 1 unadjusted for any covariate; Model 2 adjusted for baseline age, gender, BMI, peak DBP, hemoglobin, platelet count, glomerular hematuria, and uric acid; Model 3 adjusted for baseline age, gender, BMI, peak DBP, hemoglobin, platelet count, glomerular hematuria, uric acid, eGFR, and serum creatinine; and Model 4 adjusted for baseline age, gender, BMI, peak DBP, hemoglobin, platelet count, serum creatinine, uric acid, eGFR, glomerular hematuria, glomerular sclerosis ratio, segmental sclerosis ratio, and intravascular red blood cell fragments.

We investigated the impact of complement abnormalities on kidney prognosis using four adjusted models. As illustrated in Fig. 5, complement abnormality was consistently identified as an adverse factor influencing the recovery of renal function in all models. The corresponding forest plots are also presented in Fig. 5.

In addition, we performed a stratified analysis to explore the potential effect of pre-specified factors on the association between complement abnormality and renal recovery outcome. Our results showed no significant interactions between complement abnormality and various pre-specified factors such as age, BMI, hemoglobin, serum creatinine on admission, serum albumin, glomerular sclerosis ratio, and segmental sclerosis ratio (all *P* for interaction >.05) (Supplementary Table S1). However, we observed that eGFR might significantly modify the effect of complement abnormality on renal recovery this relationship (*P* for interaction = .002) (Supplementary Table S1). When eGFR ≤15 ml/min/1.73 m^2^, patients with complement abnormality were less likely to achieve renal recovery (HR, 0.117; 95% CI, 0.028–0.490; *P* = .003). While eGFR >15 ml/min/1.7 3 m^2^, complement abnormality did not associated with renal recovery (HR, 1.994; 95% CI, 0.613–6.493; *P* = .252) suggesting that complement abnormality is associated with eGFR levels (Supplementary Table S1).

## DISCUSSION

To our knowledge, the present study was the first to evaluate the complement abnormality on kidney prognosis in a large renal biopsy-proven cohort of patients with mHTN-associated TMA. Our findings indicated that complement abnormality was significantly associated with worse benefits to renal function recovery, a >50% decrease in serum creatinine from baseline, or a decrease in serum creatinine to normal, or renal survival free from hemodialysis or peritoneal dialysis for at least 1 month.

In this study, mHTN-associated TMA was defined as severe hypertension accompanied by TMA lesions observed on renal biopsies. Previous clinical investigations have explored the strong connection between malignant hypertension and TMA [4, 7, 19, 20]. Importantly, in most previous studies, patients with malignant hypertension and renal impairment were not subjected to biopsies, leading to TMA diagnosis based solely on laboratory test results rather than histopathological evidence [4, 21]. However, positive laboratory findings for TMA were rarely detected in patients with confirming malignant hypertension-induced nephropathy [21, 22]. To ensure a more representative sample, our study focused on patients with biopsy-proven malignant hypertension-associated renal microangiopathy. Within our cohort, only one patient exhibited low platelet levels (<100 × 10^9^/l), whereas thrombocytopenia reportedly affects only 24% of patients with mHTN-associated TMA [20]. Thus, a significant number of patients with mHTN-associated TMA may underdiagnosed without renal biopsy.

In patients with malignant hypertension-associated TMA, aggressive BP control can effectively alleviate acute TMA symptoms and partially restore kidney function. However, some patients fail to regain renal function despite BP control and require dialysis, particularly those with deficiencies in complement proteins [5, 11, 23]. There is growing evidence indicating the crucial role of the complement system in various clinical conditions such as immune, neurodegenerative, ischemic, and inflammatory diseases [24, 25]. Furthermore, hypertension-related renal TMA tends to recur in individuals with complement gene mutations despite aggressive antihypertensive treatment [11]. Hence, it is crucial to differentiate between TMA patients with abnormal complement activation and those without, as targeting complement abnormalities could offer therapeutic benefits [26]. For instance, landmark trials have shown that the terminal complement inhibitor eculizumab has dramatically improved the renal function of patients with TMA by blocking the formation of C5b-9 and mitigating excessive complement activation [11, 13, 27]. While exome sequencing is highly informative, it is also costly and time-consuming. Assessing plasma levels of specific complement components could be a practical and useful approach in our investigation. Notably, we observed significant diffuse deposition of complement activation products, including C3c and C5b-9, in along the arterial vasculature and/or the glomerular capillary walls, as well as in the subendothelial space. This pattern of complement deposition consistent with the findings reported by Zhang *et al*. [28] and Timmermans *et al*. [11, 27], who demonstrated the pivotal role of the alternative pathway of complement activation in the pathogenesis of malignant nephrosclerosis, as known as mHTN-associated TMA, highlighting the potential therapeutic value of targeting complement activation in the treatment of complement-mediated kidney diseases.

There is increasing evidence that the genetic mutations in complement components are pivotal in the development of hypertension-associated TMA [29]. Timmermans *et al.* [11] investigated the role of complement in nine consecutive patients with mHTN-associated TMA. In six out of nine patients, they found mutations C3 in three, CFI in one, CD46 in one, and/or CFH in two patients either with or without the risk CFH-H3 haplotype in four patients. These results confirmed that a subset of patients with mHTN-associated TMA falls within the spectrum of complement-mediated TMA, which has a poor prognosis. Consistently, Laecke *et al.* [19] reviewed the existing literature on TMA associated with malignant hypertension, emphasizing the pivotal role of the dysregulation of the alternative complement pathway in the pathogenesis of aHUS. In the context of malignant hypertension, the concomitant activation of the renin-angiotensin system and hypertension-induced shear stress in small blood vessels lead to elevated oxidative stress and pro-inflammatory responses. These factors collectively activate the complement system on endothelial cells, thereby exacerbating endothelial dysfunction. This promotes platelet aggregation and increased coagulation, further contributing to the development of TMA. Wenzel *et al.* [30] showed that that assessment of both *ex vivo* C5b-9 formation and screening for variants in complement genes may categorize patients with malignant hypertension and thrombotic microangiopathy into two different groups, including BP mediated and complement mediated. Thus, the first group requires BP control while the second group should be treated with eculizumab and BP control. In addition, it is well established that C3 plays a central role in the alternative complement pathway activation and contributes pathogenically to renal injury [31, 32], evidenced by reduced serum C3 levels, normal C4 levels, and deposition of C3c and C5b-9 along vessel walls in the present study. Our results indicated that C3c and C5b-9 were detected in the vasculature and/or glomerular capillaries in mHTN-associated TMA patients with abnormal complement levels, providing compelling evidence of the pivotal role of alternative pathway activation in the pathogenesis and progression of the disease.

Endothelial damage in severe hypertension is hypothesized to induce TMA [22]. Concurrently, hypertension-induced shear stress in small blood vessels activates complement activation on activated endothelial cells [19, 33]. Previous research indicated that this abnormal complement activation initiates inflammatory processes such as chemokine and cytokine production, adhesion molecule upregulation, and neutrophil activation. These processes exacerbate endothelial dysfunction and glomerular injury, culminating in platelet aggregation and microvascular thrombosis [27, 29, 34, 35]. Consistent with these findings, our patients with abnormal complement levels exhibited significantly lower hemoglobin, reduced platelet counts, and severe glomerular filtration impairment (mean eGFR <15 ml/min/1.73 m^2^), and lacked common hypertension-associated risk factors such as obesity, excessive alcohol consumption, and high sodium intake. Additionally, deposits of C3c and C5b-9 along glomerular capillary walls were observed in patients with abnormal complement group, confirming complement activation of the early and terminal complement pathways, respectively [36]. In conclusion, our study suggests that abnormal complement activation contributes to intravascular thrombosis, red blood cell destruction, and subsequent anemia, leading to renal dysfunction in affected patients.

Currently, there are limited population-based data on how complement abnormalities correlate with renal outcomes in patients affected by malignant hypertension-associated TMA. An analysis involving nine patients with hypertension-associated TMA revealed that these individuals exhibit characteristics of complement-mediated TMA, which typically carries a very poor prognosis [11]. Timmermans *et al*. [27] indicated that inhibiting the formation of C5b-9 could potentially aid in renal function recovery. Additionally, two studies on the efficacy of eculizumab in treating TMA associated with malignant hypertension have conflicting results [13, 37], partly due to small sample size and inadequate adjustment for critical prognostic factors such as glomerular sclerosis ratio, BMI and eGFR. By contrast, our study boasts a substantial sample size and robust statistical power. Rigorous quality control measures were employed to gather important risk factors, which were meticulously managed through multivariate and stratified analyses. Our findings confirmed a significant association between complement abnormality and poorer renal function outcomes. Notably, no statistically significant differences were observed between patients receiving conventional antihypertensive therapy. Even after adjusting for age, sex, BMI, peak DBP, hemoglobin, platelet count, serum creatinine, uric acid, eGFR, glomerular hematuria, glomerular sclerosis ratio, segmental sclerosis ratio, and intravascular erythrocyte fragments, complement abnormality remained strongly linked to renal recovery outcomes. Furthermore, activation of complement was evident in the abnormal complement group, suggesting its potential role in the pathogenesis of renal injury associated with malignant hypertension, although further validation is required.

In addition, our study revealed that eGFR played a critical role in modulating the relationship between complement abnormality and renal recovery. This was evident in both interaction and stratified analyses, particularly within the subgroup of patients with eGFR ≤15 ml/min/1.73 m^2^. This subgroup underscored the involvement of complement in the pathogenesis of glomerular endothelial cell injury. Given that eGFR serves as an indicator of kidney function, an eGFR ≤15 ml/min/1.73 m^2^ was strongly associated with ESRD, indicating near irrecoverable kidney function. Consequently, the significant association between complement abnormality and renal prognosis was primarily observed in patients with eGFR ≤15 ml/min/1.73 m^2^, where inflammatory damage led to poorer renal outcomes. Conversely, among patients with eGFR >15 ml/min/1.73 m^2^, complement abnormality did not adversely affect renal prognosis. We speculated that eGFR >15 ml/min/1.73 m^2^ being a robust predictor of renal recovery in those with mHTN-associated TMA disease, whereas nearly all patients with initial eGFR ≤15 ml/min/1.73 m^2^ progressed to renal failure.

Although the current study identified a significant association between serum complement levels and renal function recovery, several limitations should be mentioned. First, all patients in this study selected from a medical center, potentially introducing selection bias due to specific disease characteristics and/or treatment modalities unique to that institution. Although our cohort encompassed patients from The First Affiliated Hospital, Sun Yat-sen University, a comprehensive facility with diverse patient demographics, thus mitigating some selection bias-expanding the sample size is necessary to validate our findings. Second, as the current study utilized an observational design, it is important to acknowledge that residual confounding may still be present even after adjusting for a lot of potential confounders. Serum complement concentrations were only measured at baseline, overlooking potential associations between dynamic changes in complement levels and renal prognosis. Recent research has underscored the complexity of the complement system in hypertensive kidney injury, with its varying roles across disease stages [26, 38]. Moreover, our study contributes valuable data to existing literature but does not definitively establish whether complement activation is causal, a cofactor, or a consequence of severe hypertension. The diagnosis and management of renal TMA in malignancy remain challenging, necessitating further comprehensive investigations. Third, to our knowledge, the number of patients with mHTN-associated TMA included in this study represents the largest sample size reported to date. Nevertheless, it remains valuable to further expand the sample size to better evaluate the impact of tubulointerstitial damage and fibrosis on renal prognosis in patients with complement abnormalities in mHTN-associated TMA. Fourth, although our data showed that the renal tissues of in the complement abnormality group in mHTN-associated TMA patients had positive C5b-9 and C3c deposition, the interaction and histological staining of C3 and C5b-9 with serum levels of C3 deserves further expansion of clinical sample size to fully clarify. Fifth, since genetic testing was not performed, it is possible that a few cases of aHUS accompanied by high BP were included in both the normal and abnormal complement groups.

In conclusion, abnormal serum complement levels are associated with a worse renal prognosis. Patients exhibiting abnormal complement levels encounter greater challenges in achieving renal recovery. Integrating these clinical and analytical findings could enhance a more targeted approach to managing the disease based on its pathogenesis.

## Supplementary Material

sfaf235_Supplemental_Files

## Data Availability

The data underlying this article are available in the article and in its online supplementary material.
