# Supplementary material for: Complement abnormality predisposes to the development of malignant hypertension-associated thrombotic microangiopathy disease
Source: Clin Kidney J. 2025 Jul 24;18(8):sfaf235. doi: 10.1093/ckj/sfaf235 (PMC12374187; doi:10.1093/ckj/sfaf235)
Supplement: sfaf235_Supplemental_Files [file sfaf235_supplemental_files.zip › Supplementary figure legends.docx]

**Supplementary figure legends**

**Figure S1.** The scatter plots of serum C3 (a) and C4 (b) levels between the abnormal complement group and the normal complement group.

**Figure S2.** Representative immunofluorescence findings of malignant hypertension-associated thrombotic microangiopathy (TMA). (a) C3c (original magnification ×400), and (b) C5b-9 (original magnification ×400) deposits along the vasculature and/or the glomerular capillary wall in patients with localized and segmental complement abnormalities.
