# Supplementary figures and images for: Complement abnormality predisposes to the development of malignant hypertension-associated thrombotic microangiopathy disease
Source: Clin Kidney J. 2025 Jul 24;18(8):sfaf235. doi: 10.1093/ckj/sfaf235 (PMC12374187; doi:10.1093/ckj/sfaf235)

**a**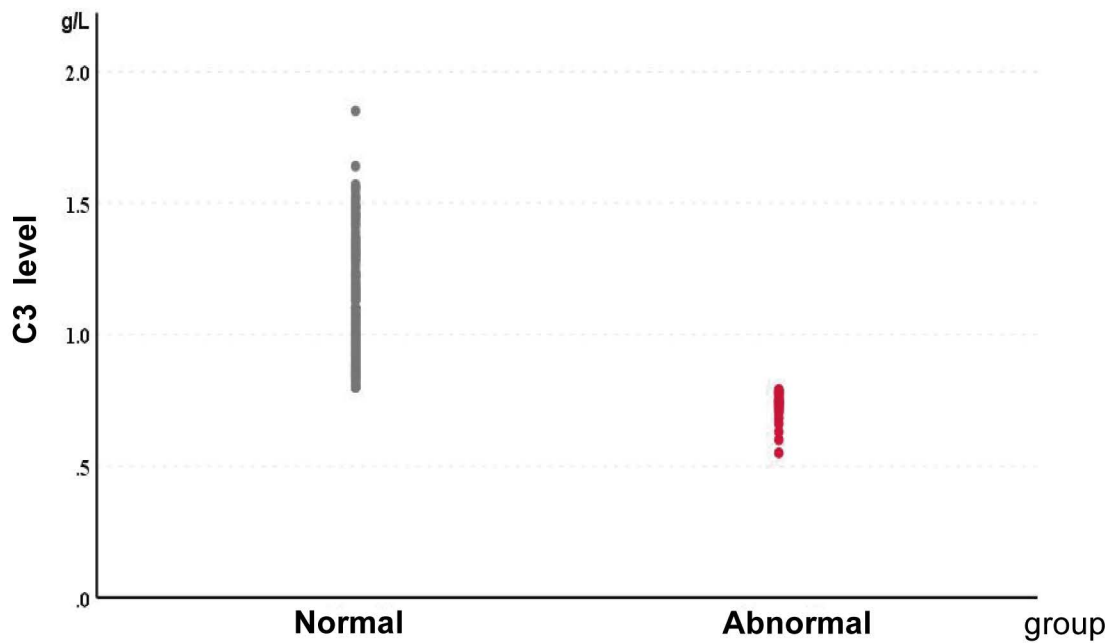**b**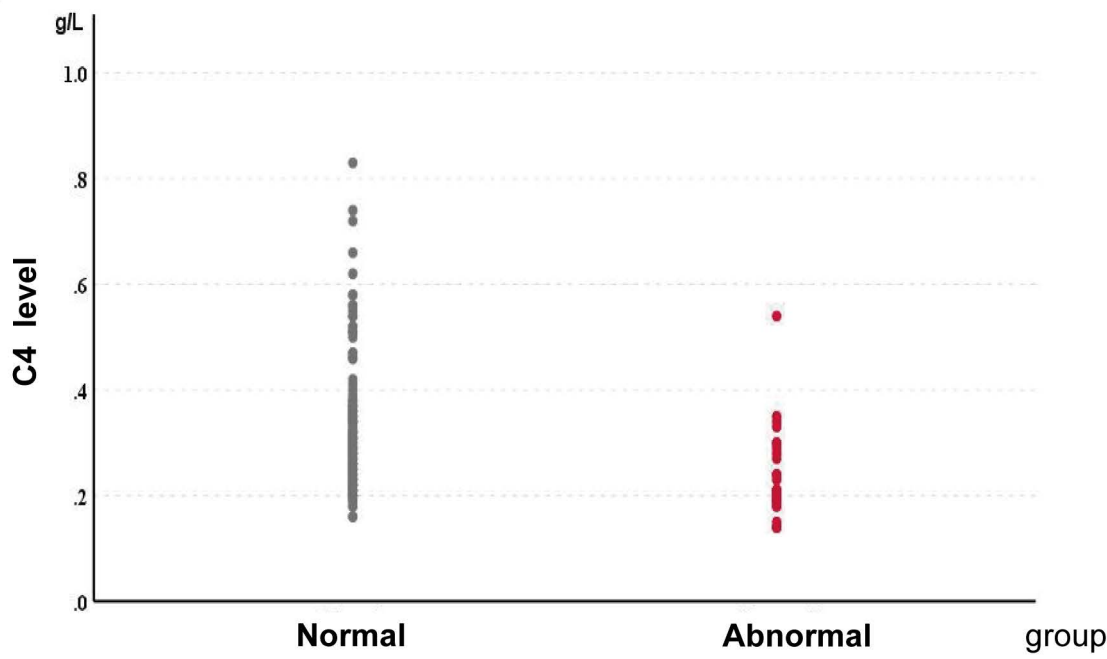

Supplement: sfaf235_Supplemental_Files [file sfaf235_supplemental_files.zip › Figure S1.pdf]

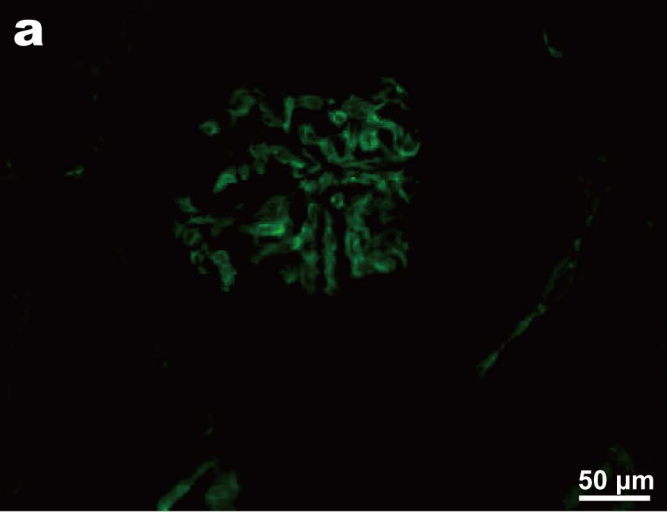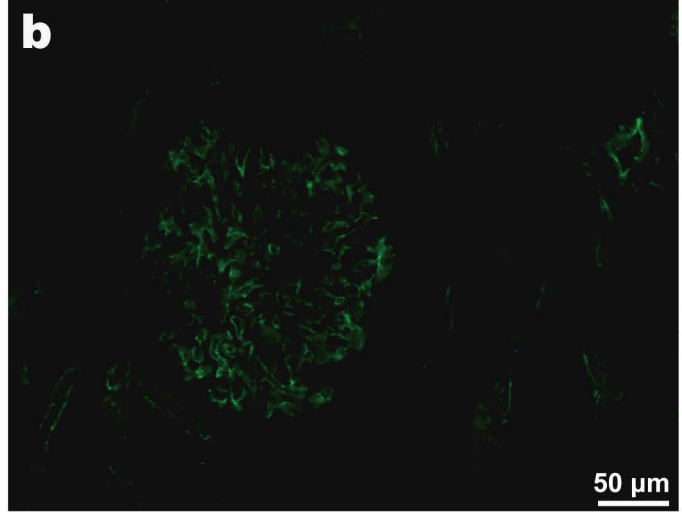

Supplement: sfaf235_Supplemental_Files [file sfaf235_supplemental_files.zip › Figure S2.pdf]
